# The Impacts of Colony Cages on the Welfare of Chickens Farmed for Meat

**DOI:** 10.3390/ani12212988

**Published:** 2022-10-30

**Authors:** Jenny L. Mace, Andrew Knight

**Affiliations:** Centre for Animal Welfare, Faculty of Health and Wellbeing, University of Winchester, Sparkford Road, Winchester SO22 4NR, UK

**Keywords:** broiler chicken, meat chicken, chicken behaviour, animal welfare, housing type, modern colony cage, litter floor

## Abstract

**Simple Summary:**

Over 70 billion chickens are slaughtered globally each year. Almost all are meat breeds, typically housed in very large barns with a litter floor. Recently however, modern cage systems have been developed which provide very limited space and stack several tiers high. There is debate about the impacts of such modern cage systems on chicken welfare. Accordingly, we systematically reviewed studies assessing the welfare of meat chickens kept in either modern cage systems or littered barns. Overall, 23 studies were reviewed, and none of the experimental studies reviewed incorporated a full behavioural analysis. Therefore, significant concerns exist about the deprivation of natural behaviours in meat chickens kept in modern cage systems. Given the numbers of meat chickens globally that could be impacted by these modern cage systems, this is a major animal welfare concern. Instead of implementing such systems, research and development should focus on improving the welfare of meat chickens kept in littered barns. A full behavioural analysis—as included in gold-standard animal welfare assessments, such as the Welfare Quality Assessment protocols—should form a mandatory part of any future studies investigating the welfare impacts of housing systems on chickens.

**Abstract:**

There is growing interest in keeping meat chickens in modern colony cages (CCs) rather than conventional litter-floor barns. Suggested welfare improvements for chickens in such systems include reduced bodily lesions due to lower contact with flooring contaminated with faeces and urine, due to slatted flooring and automated faeces removal. This systematic review sought to determine the animal welfare impacts of CCs using slatted flooring, in comparison to litter-based non-cage systems. Overall, 23 relevant studies were retrieved. From one perspective, the extant research appeared mixed. Fifteen (65%) of these 23 studies identified some form of welfare concern about slatted floors, and thus CCs. Yet, when considering actual welfare indicators assessed, the tallies generated in favour of each housing system were similar. Crucially however, there were incomplete behavioural welfare measures in 100% of the empirical studies reviewed. Accordingly, significant welfare concerns exist about CCs, centring around behavioural deprivation. Given that over 70 billion chickens are farmed then slaughtered each year globally, widespread implementation of CCs would create a major animal welfare concern. Instead of implementing such CC systems, research and development is recommended into improving welfare outcomes of conventional litter barns using different forms of commercially feasible enrichment. As a minimum, a full behavioural analysis, as detailed in the Welfare Quality Assessment protocols, should form a mandatory part of any future studies aimed at assessing the welfare impacts of housing systems on farmed chickens.

## 1. Introduction

**Figure 1:**
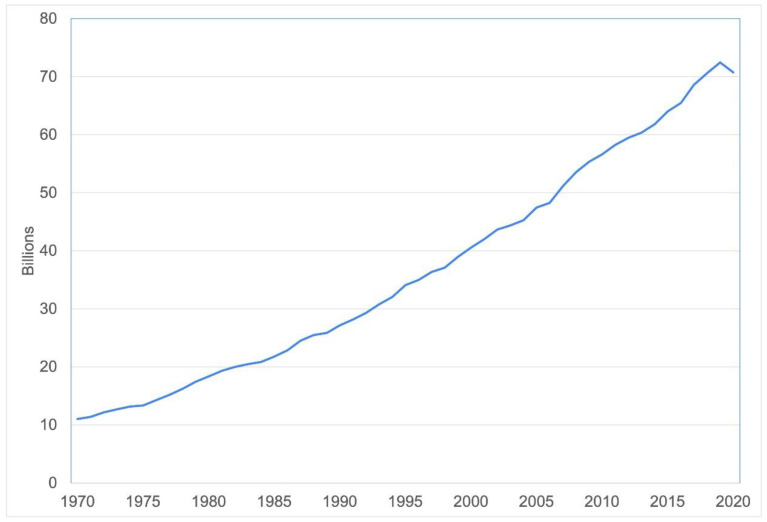
Chickens slaughtered globally. Data source: [1].

Chickens are the most intensively farmed terrestrial animal species. From 1970 to 1990, the global population of chickens alive at any time during each year doubled. This further tripled over the next thirty years, exceeding 33 billion by 2020. Most are meat breeds slaughtered after only a few weeks, rather than laying hens. Hence, many more chickens are slaughtered annually, than the living population. Over 71 billion chickens were slaughtered annually by 2020 (Figure 1) [1].

To maximise efficiencies of production, minimising feed, housing and management costs, most poultry production systems have relied on large group sizes and high stocking densities—typically with thousands of birds housed within barns on deep litter systems [2]. Recently however, some industry suppliers have developed multi-tier colony cages (CCs) for growing meat chickens. The Big Dutchman company, for example, markets CCs designed to house 60–120 growing chickens, in 50 cm high cages. The cages are designed to stack vertically (designs are provided for systems four tiers high, although some farms may use additional tiers as noted below), yielding “two to four times higher stocking density as compared to floor production” [3]. Cages are designed with front-opening panels facilitating removal. The increased production efficiencies offered by such systems could stimulate their widespread adoption, transforming meat chicken production globally. However, in light of the numbers of chickens potentially affected, this could have major implications for animal welfare.

The litter-free plastic flooring within the Big Dutchman CCs is slatted, allowing manure penetration onto plastic sheets for later removal. The company claims this system increases hygiene and reduces welfare problems such as feet, breast, and skin injuries, and infections [3]. However, such systems further inhibit the ability of chickens to fulfil highly motivated behavioural needs, compared to chickens raised in barns, such as foraging, dustbathing, and perching [2]. Accordingly, concern exists about the impact of such systems on poultry welfare. 

To assess such impacts, in 2020 the Israeli Ministry of Agriculture and Rural Affairs examined the welfare of meat chickens raised within CCs compared with litter-based systems, on two Israeli farms. One hundred randomly selected birds were examined on farm and after slaughter. The litter-based system resulted in lower hock burns, higher ease of movement, and better thermal comfort (no panting was observed). The CC system had superior nipple drinker spacing and resulted in greater plumage cleanliness. However, the authors noted that behavioural examination was limited, preventing the correlation between all parameters necessary for adequate welfare assessment. They concluded that further research was necessary, to compare welfare between traditional deep litter and CC systems [4].

However, significant relevant research already exists, which can provide insights into the likely welfare implications of CC systems for farming meat chickens, compared to litter-based systems. Accordingly, we conducted a systematic review to identify and evaluate studies of the welfare impacts of raising meat chickens within CC or litter-based systems. Cage systems invariably included slatted flooring, allowing manure egress. Such slatted flooring can create risks of abrasions, skin, foot, and leg injuries. Accordingly, we also aimed to identify and consider studies of the welfare impacts of slatted flooring. 

## 2. Methods

Consistent with systematic review guidelines [5], our aims were to identify, evaluate, collate, and analyse all good quality studies aimed at providing insights into the welfare impacts of caged or slatted floor housing systems for meat chickens, in comparison to litter-based systems.

Two bibliographic literature databases were chosen for this purpose. Scopus is one of the world’s largest databases covering the life and health sciences [6], and the only major professional life sciences database accessible to us at the time of our survey. Accordingly, we used this database, along with Google Scholar. The latter is freely accessible and is estimated to include approximately 80–90% coverage of all articles published in English, totalling nearly 100 million documents by 2014 [7].

In January 2021, we inputted the following search terms into both databases: broiler chicken housing systems, broiler chicken slatted floor, broiler chicken slatted floor welfare, broiler chicken flexible plastic netting, broiler chicken CCs, or broiler chicken welfare cages. We chose ‘broiler chicken’ as it is the terminology most commonly used to describe chickens raised for meat (e.g., [8]), although we use the term ‘meat chicken’ throughout this article, partly as the cooking methods used are not limited to broiling. Due to significant genetic and anatomical differences between modern-day laying hens and meat chickens, as well as significantly different durations spent on farms, we excluded any studies that focused on laying hens. 

Following each database search, the first 10 pages of results were examined for relevant studies. Any focusing solely on measuring performance or production indicators, rather than animal welfare, were excluded. If a whole page of non-relevant results resulted prior to 10 pages, searches using that particular search phrase were terminated at that point. 

There were no restrictions applied to the methodology used in the studies. All types of litter floor and all types of cage and slatted floor were included, as there were often key similarities to modern CCs, allowing reasonable extrapolations regarding certain aspects of welfare. Slatted floors are used in all cage types to allow manure egress, but are not universally limited to cages. Hence, we simply referred to ‘slatted floors’ when describing studies of slatted floors. However, the welfare impacts of slatted floors in non-cage systems are also relevant to cages. We specified the exact nature of the housing system where relevant to the welfare indicators assessed. 

Akin to the approach of Riber et al. [9], studies published prior to 2000 were excluded due to the diminishing relevance of results to modern-day chickens, who continue to be selectively bred for rapid growth and body weight gains, becoming ever more anatomically distant from chickens of prior decades. Papers in languages other than English were also excluded.

The retrieved studies were distributed into tables according to their findings and welfare indicators assessed. We then analysed their findings, and their validity for the assessment of chicken welfare. 

Some variations exist in conceptualisations of animal welfare, and throughout this review we have used an up-to-date, simple, and widely adopted definition of animal welfare, namely “the physical and mental state of an animal in relation to the conditions in which it lives and dies” [10,11].

## 3. Results and Discussion

Our systematic review retrieved 33 relevant papers. Nearly all were peer-reviewed articles published in academic journals; however, two post-graduate research theses were also included. The term ‘papers’ or ‘studies’ is used to refer to all retrieved articles or theses. These papers are summarised in Table 1, Table 2, Table 3 and Table 4, as:

Table 1: Papers suggesting at least some form of welfare problem using slatted floors or cages

Table 2: Papers suggesting either welfare advantages from slatted floors or cages, or no significant differences between floor types 

Table 3: The health/welfare indicators used in all papers in Table 1 and Table 2 and their association with floor type 

Table 4: Papers suggesting alternative commercially feasible methods of improving common litter-reared meat chicken welfare problems

Key findings from these papers are discussed in the following subsections. First, general characteristics of the studies are described. Next, welfare comparisons between slatted floor and litter-based systems are drawn. Following this, weaknesses of the extant research are discussed, and welfare justifications for modern CCs and slatted flooring systems are examined and critiqued. Finally, we consider possible alternative research directions, and strategies for advancing animal welfare.

### 3.1. Study Characteristics

The number of studies directly comparing some welfare parameters of meat chickens reared on litter floor variations with those raised on slatted floor variations totalled 23 (Table 1 and Table 2). All were published from 2000 onwards, with 17 (74%) published between 2015 and 2020. Four (17%) explicitly referred to equipment from the aforementioned Big Dutchman company [13,17,20,33], though these studies did not test any kind of cage, but simply a fully slatted floor. Another study mentioned similar equipment from the company Kutlusan [22]. Four (17%) of the 23 studies described the slatted floor used in their study as “mesh”, “flexible”, “cushioned”, or as a “net” [12,19,22,26]. Some studies did not give caging details such as origin/design, size, or construction material (e.g., [21,25,28]. Many of the studies utilised a slatted floor, but some were unclear about whether these were soft or flexible (e.g., [18,29]). 

Of the 23 studies comparing litter floors with some form of slatted floor, 14 (61%) were conducted in hot climates such as Egypt, Turkey, Nigeria, China, and Indonesia. Four (17%) were conducted in the USA (one), or Germany (three). Four others (17%) were international review studies, and one (4%) was an international expert survey. Of the 18 empirical (non-review) studies, welfare measures were based on physiological and behavioural parameters/tests, as summarised in Table 3. Additionally, 13 studies used fast-growing meat chicken breeds (Ross 308/PM3 or Cobb 500); three used a slow-growing breed (Hubbard); two used a mix of the aforementioned fast- and slow-growing breeds; and two used a “white-feathered” chicken with further details absent. The stocking densities of the chickens ranged from 10–27 birds/m^2^. In all studies, the chickens originated from commercial hatcheries and arrived at experimental setups as day-old chicks. All studies controlled for environmental variables such as ventilation and food, typically feeding commercial feed, and following guidelines for commercial breeds (e.g., [8]). Thus, there was partial uniformity between the studies in these regards.

### 3.2. Welfare Comparisons between the Studies

Overall, 15 (65%) of the 23 studies (Table 1) provided evidence of welfare compromises that could result in a slatted floor system, whilst eight (35%) of the studies (Table 2) suggested either welfare advantages from slatted floors, or no significant difference in welfare between floor types. It is important to note that some of the aforementioned 15 studies indicating certain welfare compromises in slatted floors, also indicated welfare improvements in some other parameters. Indeed, when considering a tally of the individual welfare indicators (rather than the number of studies suggesting any welfare compromise) as pooled in Table 3, the mixed nature of the evidence becomes clear: within the 23 papers, there were 29 indicators of better welfare in conventional litter systems, 31 indicators of better welfare in some form of slatted floor, and 27 indicating no significant welfare differences between litter and slatted floors. 

The papers in Table 3 are also spread fairly evenly across the various welfare measures, suggesting evidence across a broad range of welfare parameters for/against/neutral with respect to slatted floors. However, Table 3 does indicate one key area in which evidence is lacking for welfare enhancements in slatted floors—behavioural indicators. 

From a welfare perspective, neither welfare advantages from slatted floors, nor a lack of significant welfare differences between slatted and litter floors, would prove an obstacle to transitioning to slatted floors. Combining the relevant columns (‘Slatted floor best’ and ‘No significant difference’) in Table 3 yields 58 instances of welfare indicators within studies that suggested no welfare compromises from slatted floors, compared to 29 suggesting welfare would be compromised. However, behaviour was included in only four studies (and none of these fell amongst the studies suggesting slatted floors produced better welfare), despite best practice stipulating that animal behaviour should be a major component of animal welfare assessment [44]. Additionally, a cage environment—even a modern CC—offers fewer possibilities to improve welfare through environmental enrichment.

Two of the four systematic reviews to date have recommended against cages [16,23]. The two other reviews concluded there were no significant differences in welfare between housing types [30,32]. The review by Sargeant et al. [30], p. 247, however, also emphasised the “poor reporting of key design features in many studies, and analyses rarely accounted for non-independence of observations”. The review by Baracho et al. [32] missed out numerous relevant studies, covering only two that included housing type and dedicating only one small paragraph to discussion of housing/floor type. The expert survey by Bracke et al. [15], using a semi-quantitative and semantic modelling approach, found all major conventional intensive housing systems including modern cage systems to be unacceptable in terms of meat chicken welfare. This was based on both input and output welfare measures.

### 3.3. Could Colony Cages with Slatted Floors Improve Chicken Welfare?

Amongst the 23 cited studies in Table 1 and Table 2, the most similar housing type to that of modern CCs featured in the study by Şimşek et al. [22]. The modern CC used in this study was designed by the company Kutlusan [45]. It incorporated automatic faeces removal via conveyor belt, automated chicken movement to transportation crates (for onward transport to slaughter) also via conveyor belt, and a flexible plastic mesh slatted floor. This Turkish study found significantly higher serum malondialdehyde levels—indicative of stress—in chickens in the CCs, than in those reared on litter floors. It also found more wing/breast bruising and wing fractures in the cages, which the authors attributed to the automated conveyor belt process transporting chickens to collection crates. The study did also find higher footpad lesions in litter floor systems, but this welfare issue was far from absent in the cage system. In contrast to the majority of other studies in Table 1 and Table 2, this study also found better performance indicators in chickens reared on litter.

On the face of it, several factors initially appear to indicate that CCs with slatted floors could improve, or at least not decrease, meat chicken welfare: (1) some evidence suggests welfare improvements relating to footpad dermatitis (FPD), hock burn, and breast blisters, especially using soft, flexible slatted floors compared to more abrasive materials such as hard plastic, wire, or wood—although evidence is mixed; (2) ‘touch-free’ automated onward movement systems could decrease handling stress; and (3) the shortened lifespans of meat chickens until slaughter (around 35 days is common), can lower cumulative welfare impacts, compared to longer-lived chickens. These factors will be considered in turn.

First, claims exist of increased incidence of FPD, hock burn, and breast blisters in littered barns, relative to slatted floors [3,46]. This can result from greater bodily contact with contaminated flooring in littered systems due to increasing contamination with faeces and urine over time, and increasing immobility as chickens gain weight. It is true that these are pressing welfare concerns [2]; however, there is a lack of consistent evidence for improvements of these welfare problems when using slatted floors. Table 3, for instance, shows a comparable number of studies simultaneously claiming (1) litter to be the best floor type for avoiding FPD, breast blisters, and/or hock burn; (2) a slatted floor to be the best floor type for avoiding these same conditions; and (3) no significant differences in the prevalence of these same conditions between different floor types. 

Evidence is also mixed regarding whether using a soft and flexible slatted flooring material will overcome FPD, hock burn, and breast blister risks. Of the four studies that made their use of a flexible material explicit, all are in Table 1, showing superior welfare outcomes when using a litter floor. However, one did not examine FPD, hock burn, or breast blisters formally [26], one did not compare slatted floor with litter [12], one found higher FPD in litter [22], and one found a significantly lower prevalence of breast blisters in cages, but did not find the same for FPD [19]. Investigators also highlighted concerns with higher incidence [42] or at least an ongoing significant prevalence of FPD in slatted floors. For example, Çavuşoǧlu et al. [18], p. 13 reported a 20% prevalence of FPD in slatted floors. Considering these mixed effects of slatted floors on FPD, hock burn, and breast blisters, and other key welfare concerns independent of slatted flooring material, such as behavioural deprivation, it is arguably a distraction to overall considerations of welfare, to focus overly on the effects of slatted floor systems on FPD. 

Second, justifications for modern CCs have also been based on claims of decreased chicken stress resulting from ‘touch-free’ transfer from CCs to transport crates via conveyor belts [3,46]. The importance of this should not be underestimated as catching is considered one of the most stressful processes for chickens. However, in these automated modern CCs, the handling time is only reduced, not eradicated, with chickens still being handled afterwards when moving them from the table to transport crates [47]. Our systematic review did not identify any studies directly focused on the welfare implications of such automated systems in modern CCs. However, Simsek et al. [22] postulated that the automated conveyor belt system forwarding chickens to transportation could be the cause of the increased wing fractures, and wing and breast bruising, identified in their study. Additionally, there are other automated initiatives that have been studied that could be used in litter floor systems, such as the Apollo Generation 2 chicken harvester [48,49,50]. Importantly, evidence for clear welfare advantages of automated systems relative to the more traditional abdomen-upright manual catching method appears to be lacking, with welfare levels dependent on factors such as line speed, handler training and handler disposition [49,50]. What is clear is that manual catching of chickens via one leg and carrying them in an inverted position risks injuries, is extremely stressful, and should be prohibited on welfare grounds. Simsek et al. [22] have also found a link between this catching method and increased carcass bruising. Norway has prohibited this practice. Kittelsen et al. [51] even suggested that the abdomen-upright catching method can be faster than catching chickens by the feet.

Thirdly, with respect to the shortened lifespans of meat chickens prior to slaughter, compared to longer-lived animals, Nagar and Dov [46] noted that meat chickens are relatively immobile by six weeks of age (slaughter normally occurs at or before this age), arguing that their welfare may therefore not be very compromised relative to laying hens in cages. However, anecdotal evidence from those rescuing ex-meat chickens suggests that, with modified husbandry practices, meat chickens can walk, jump, and even fly at ages well surpassing industry slaughter ages [52]. Thus, there needs to be an emphasis on how to increase and optimise movement of meat chickens through nutritional, enrichment, and other husbandry improvements, as they are not irrevocably destined to immobility by six weeks of age. 

### 3.4. Limitations of Retrieved Studies

#### 3.4.1. Welfare Indicators Assessed

One salient weakness of the 23 studies retrieved is indicated by the fact that only nine (39%) actually mention welfare in their titles, seven of which are located in Table 1 (indicating welfare problems using slatted floors or cages). This suggests animal welfare was not the primary focus of most retrieved studies, in contrast to non-welfare related foci. Similarly, the Welfare Quality Assessment (WQA) protocol was recently described as the most comprehensive scientifically validated means of measuring chicken welfare [44]. This has been developed in collaboration with multiple stakeholders including farmers, and both animal-based and input measures are incorporated within the WQA [53]. Yet, only six (33%) of 18 retrieved empirical studies mentioned the WQA explicitly, with one other mentioning an ‘ethogram’ and another mentioning RSPCA welfare measures. The expert survey also refers to WQA. 

Of these six studies, none consider the WQA in full. Instead, they take only a partial approach. For example, Li et al. [19] include only eight of the 12 WQA criteria and all studies exclude qualitative behavioural analysis (QBA), which forms a key component of the WQA. The WQA does state that some measures—such as “cover range” (p. 30) under the criterion “expression of other behaviours”—are not applicable to non-free-range systems, but this does not apply to QBA (p. 31). Moreover, the WQA states that under such circumstances a score of “zero” should apply to measures not applying to barn/cage systems (p. 30), meaning these measures should not just be left out or ignored—as normally occurred in these studies. 

This incomplete application of the WQA occurred despite the fact that one of the greatest welfare concerns for caged chickens is behavioural deprivation, and the resulting negative emotional state this may bring—aspects that the QBA indicator of the WQA aims to capture. Chen et al. [12] stated, “it is impossible to observe and evaluate broilers in dim, restricted, and high-density cages … [t]hus, the assessment of qualitative behaviors was cancelled in this research” (p. 3). Yet, the WQA (p. 23), explicitly states that lights should be used to help with such measurements. The use of cameras could also help [54]. At least, these measures should be included and scored as zero if conditions result in visibility so poor that bird behaviour cannot be assessed. As an example, a more accurate application of the WQA to which these studies can be compared, is provided by Gocsik et al. [55]. This, for example, includes QBA, and notes that QBA outcomes are affected by stocking density, enrichment, and flock size.

Within the eight (44%) of the 18 empirical studies retrieved that did measure some form of behaviour, this was limited to a very narrow range—such as walking ability alone in the study by Çavuşoǧlu et al. [18]. No studies measured dust-bathing or foraging behaviour, or vacuum behaviours (indicative of stress)—not even in a study that included a dust bath in its slatted floor test setup [33]. Chicks are known to dust bathe from as young as a few days old [56]. There is no mention of dust baths in the Big Dutchman modern CC brochure examined [3]. This leads to further concern regarding likely behavioural deprivation that could ensue in modern CCs. 

The most comprehensive behavioural measures were actually recorded by Fouad et al. [24] and Fortomaris et al. [25]. Ironically, these studies did not claim to use the WQA. Moreover, they are two of only four studies that combined behavioural and physiological methods, which is deemed to provide the most accurate welfare assessment results, due to compensation for the weaknesses of each method of welfare assessment, by the other [44]. Crucially, none of the studies in Table 2 (supporting the use of slatted floors) combined both behavioural and physiological aspects. Ten (43%) of 23 studies restricted their investigations to physiological and other non-behavioural parameters, or focused on only one particular aspect of welfare, such as footpad health (e.g., [33]). There is thus an incomplete picture of welfare in all of the studies to varying degrees, and conceivably, if welfare were measured in a more thorough and holistic fashion, more studies would favour conventional litter floors, which offer greater opportunities to exercise important natural behaviours such as dust-bathing, foraging, walking, and wing-flapping. 

Stocking density is another key factor influencing environmental complexity, the ability of chickens to express highly motivated behaviours, and stress levels. In their brochure, Big Dutchman recommend stocking densities of 50 kg/m^2^ [3]. This would be illegal in the EU, which has maximum stocking densities of 33–39 kg/m^2^ [57]. It would also be higher than used in litter floor systems. This recommendation is also contrary to recent research highlighting improved welfare when stocking densities are lower than 38 kg/m^2^ [58]. Moreover, only three studies [14,25,28] employ stocking densities comparable to current industry norms worldwide of around 42 kg/m^2^ [59], limiting the applicability of study results to the prediction of meat chicken welfare on commercial farms.

#### 3.4.2. Inconsistencies in Study Design and Reporting

Additional weaknesses among the retrieved studies relate to study design and reporting. Several aspects such as stocking density, caging materials, enrichment means (if any), and welfare indicators assessed, were far from uniform among the studies. This lack of consistency in approach is problematic, and was also highlighted in another systematic review [30]. Such variation in variables that could potentially affect results may well explain some of the observed heterogeneity within the results. There is also a paucity of relevant information in some studies, such as that by Özhan et al. [21], which does not provide any details about cage design. Another concern was that three studies employed a higher stocking density for their cage experimental setup, than for their litter floor setups [14,24,25]. This reduces the validity of subsequent comparisons between housing types. It is also recommended to use ‘birds/m^2^′ as the stocking density unit rather than ‘kg/m^2^′ as the weight of the chicken changes quickly throughout the rearing process. Some studies also removed accumulating faeces (e.g., [22]), whilst others did not (e.g., [19]).

#### 3.4.3. Studies Favouring Slatted Floors

The research design flaws highlighted above were frequent among the eight studies indicating that slatted floors or cages generally did *not* create welfare concerns (Table 2). Despite its importance within welfare assessment, only the study by Çavuşoglu and Petek [29] considered behavioural outcomes. Another admitted that behavioural measures would have been beneficial [33]. Additionally, despite this being best practice as noted previously, no studies combined both behavioural and physiological parameters. One study [27] did not specify the stocking density. Another used a different breed to all other studies—the New Lohmann breed [34]. Two had a very narrow scope, limiting wider applicability of their results [31,32]. One review by Sargeant et al. [30] highlighted the poor reporting of research design features, and the lack of accounting for non-independent assessors. 

### 3.5. Future Research to Improve Farmed Chicken Welfare 

The welfare of meat chickens in current systems is threatened by multiple factors—notably behavioural, environmental, and spatial restrictions [2], and is accordingly, often poor. Given this, the 27 instances of welfare indicators which were not significantly different between cage/non-cage systems (Table 3), cannot be interpreted as an endorsement of modern CCs. In fact, the spatial and environmental restrictions inherent within CCs limit their capacity for welfare improvements—notably, in the key behavioural domain of welfare. Fortunately, however, we identified 10 studies (Table 4) suggesting commercially feasible means of overcoming common welfare concerns arising within conventional littered barns, including those relating to breast blisters, hock burn, and FPD. A few studies in Table 1 and Table 2 also incorporated such measures. These included increasing the distance between resources/enrichment [38], as well as the provision of platforms rather than perches [40] and laser lights [39] as forms of environmental enrichment to encourage movement and a wider variety of highly motivated behaviours, lowering contact time with the litter. Floor heating [13] to help dry litter, regular replacement of the litter [37], and using peat in preference to traditional litter materials [40] have also been suggested to lower infection risks. Several of these studies (e.g., [37,39]) also indicated the implementation costs of such initiatives were low. Some, such as the study by Simsek et al. [43], even suggested possible benefits for performance (i.e., production parameters), and thus economic viability. 

In light of the well-established evidence for behavioural deprivation that would ensue in modern CCs, and the possibility of better performance in litter-based systems (e.g., as suggested by Simsek et al. [43]), further research aimed at improving farmed chicken welfare would best be directed into developing such technological and management solutions to common welfare problems in litter systems, including the use of cameras to monitor behaviour [54,60,61]. Additionally, given its importance as a welfare domain, future research aimed at assessing welfare should normally include a thorough behavioural analysis.

## 4. Conclusions

In conclusion, 65% of the 23 most relevant studies retrieved and reviewed raised some form of welfare concern about slatted floors, and thus about modern CCs. Moreover, 100% of the empirical studies reviewed utilised incomplete behavioural analyses (if these were used at all). Thus, significant welfare concerns exist about modern CCs, centring around behavioural deprivation. Furthermore, their design constraints severely limit their potential for overcoming this problem. Additionally, a full behavioural analysis, as detailed in the Welfare Quality Assessment, should form a mandatory part of any future studies aimed at assessing the welfare impacts of housing systems on chickens.

## Figures and Tables

**Table 1:** Studies (15) suggesting at least some form of welfare problem using slatted floors or cages. Nb: If the material of the slatted floor was not stated, it was not reported. Stocking densities were not converted into consistent measures due to variable chicken body weights. WQA refers to Welfare Quality Assessment. Studies are sorted by publication date.

No.	Author(s); Date	Research Design	Title/Key Research Question	Sample				Key Result(s) and Recommendation
				Country; Sample Size	Floor/Housing Types	Stocking Density	Chicken Breed/Type	
1	Chen et al. (2020) [12]	Welfare evaluations of broilers using WQA	Comparison of Chinese broiler production systems in economic performance and animal welfare	China; 66 flocks on 52 farms	(1) Net floor (NF) (2) Normal cage (NC) (3) High standard cage (HSC) (Iron cages)	Variable stocking densities in the NF. NC/HSC = >50 kg/m^2^	White-feathered	Welfare decreased over time amidst shift from NF to NC, and from NC to HSC. An overall negative correlation was found between welfare and profit. Net floor may be preferable to colony cages. Nine out of fourteen measures showed significant differences between the three systems.
2	Abd El-Wahab et al. (2020) [13]	Dissection	The effects of feed particle size and floor type on the growth performance, GIT development, and pododermatitis in broiler chickens	Germany; *n* = 480	(1) Wood shavings (2) Wood shavings + heat (3) Partial slat (4) Plastic slatted floor (Plastic covered steel; width 3.5 mm, Big Dutchman)	20 birds/m^2^ pens	Ross 308, both sexes	Using a fully slatted floor led to a higher body weight while having no effect on reducing the incidence of footpad dermatitis. Housing birds on litter with floor heating resulted in the lowest pododermatitis prevalence.
3	Abo Ghanima et al. (2020) [14]	Physiological measures	Impact of different rearing systems on oxidative stress biomarkers	Egypt; 270; 12 birds per group	(1) Litter—wood shavings, 5cm depth (2) Perforated plastic slate, steel bars covered with plastic (3) Cage, steel batteries with dimensions of 100 × 90 × 45 cm	12 birds/m^2^ (litter) 12 birds/m^2^ (perforated) Not stated (cage)	Cobb	Higher oxidative stress was found in cage systems. Eosinophil, lymphocyte, basophil, and monocyte counts, and phagocytic index and activity were reduced in litter systems.
4	Bracke et al. (2019) [15]	Expert survey	Broiler welfare trade-off: A semi-quantitative welfare assessment for optimised welfare improvement based on an expert survey	International; 20 meat chicken welfare scientists and 7 veterinarians	n/a	n/a	n/a	It appeared that experts use both input and output parameters to explain overall welfare, and that both are important. The major conventional systems and modern cages for meat chickens received low welfare scores, well below scores that may be considered acceptable.
5	El-deek et al. (2019) [16]	Review	Behaviour and meat quality of chicken under different housing systems	international	n/a	n/a	n/a	Housing system, as a non-genetic factor, directly affects the welfare of the birds and can impact their behaviour. Free-range production system might be considered a favourable alternative housing system. The majority (approximately 70%) of intensive production systems that are currently used do not usually support the natural behavioural needs of poultry.
6	Almeida et al. (2018) [17]	Behaviour and physiology, WQA	Poultry rearing on perforated plastic floors and the effect on air quality, growth performance, and carcass injuries—Experiment 2: Heat stress situation (part 1 in no. 9 below)	Brazil; *n* = 384	(1) Perforated plastic (faeces removed), Big Dutchman (2) Wood shaving (heat stress)	12 birds/m^2^	Cobb 500, mixed sex	Use of perforated plastic flooring in a heat stress situation can improve air quality (less CO2 and NH3 concentration) and bird cleanliness. On the other hand, chickens are more susceptible to develop lesions in the breast, hock, and footpad. More research is required into bird wellbeing.
7	Çavuşoǧlu et al. (2018) [18]	Animal-based welfare parameters were measured, WQA	Effects of different floor housing systems on the welfare of fast-growing broilers with an extended fattening period	Turkey; *n* = 210	(1) Deep litter (2) Litter and slat (3) Slatted floor	Not stated	Male fast-growing hybrids, Ross PM3	Haemorrhage or lesion scores of the breast and shoulder of broilers with slat floor housing were found to be significantly greater than for conventional deep litter as a result of a heavy body weight at a greater slaughter age.
8	Li et al. (2017) [19]	WQA	Effects of two different broiler flooring systems on production performances, welfare, and environment under commercial production conditions	China; four flocks, 31,700 per flock	(1) Litter—rice hulls, 10 cm (2) Perforated plastic floor (3) Netting—faeces not removed	Meat chicken house = 18 × 150 m, 31,700 per house 12 birds/m^2^	Cobb 500	The average ammonia concentration was lower at 10.44 ppm in the litter house compared to 15.02 ppm in the netting flooring house due to manure accumulation under the floor. Birds raised in the netted floor house had increased breast blister incidence. However, no difference was found in foot/hock lesions, lameness, and fearfulness.
9	Almeida et al. (2017) [20]	WQA and injuries	Poultry rearing on perforated plastic floors and the effect on air quality, growth performance, and carcass injuries—Experiment 1: Thermal Comfort (part 2 in no. 6 above)	Brazil; *n* = 384	(1) Litter—wood shavings turned frequently (2) Perforated plastic floor (identical to pig maternity floor), faeces removed	12 birds/m^2^	Both sexes, Cobb	The highest scores of footpad dermatitis were found in the slatted case.
10	Ozhan et al. (2016) [21]	Blood samples, other physiological measures	Comparison of floor and cage housing systems in terms of some welfare assessments in broiler chickens	Turkey; *n* = 30	(1) Floor (2) Cage	Not stated	Ross 308	Cage housing system negatively affected broilers’ blood parameters, bone quality, and pH level of breast muscle.
11	Simsek et al. (2014) [22]	Physiological parameters	Effects of cage and floor housing systems on fattening performance, oxidative stress and carcass defects in broiler chicken	Turkey; *n* = 30	(1) Wood shaving (2) Four storey cage unit—base of each storey was made from plastic mesh material. Faeces removed via conveyor belt (Kutlusan)	17–17.5 chicks/m^2^	Ross 308 broiler	Cases of wing fractures and wing and breast bruising were found to be higher with cage housing. Serum malondialdehyde level increased with cage housing. The results of this study indicated that floor housing had shown better performance and carcass quality at the production capacities examined.
12	Shields and Gregor (2013) [23]	Review	Animal welfare and food safety aspects of confining broiler chickens to cages	International	n/a	n/a	n/a	Cage environments are usually stocked at a higher density than open floor systems, and the limited studies available suggest that caging may lead to increased levels of fear and stress in the birds. Further, birds reared on the floor appeared less likely to harbour and shed Salmonella, as litter may serve as a seeding agent for competitive exclusion by other microorganisms. Cages likely to meet with public disapproval.
13	Fouad et al. (2008) [24]	Behavioural and physiological parameters, ethogram	Broiler welfare and economics under two management alternatives on a commercial scale	Egypt, 2 × 12,375 flocks	(1) Litter—10 cm wood shaving (2) Cage—three vertical tiers, six per cage	Floor: 16 bird/m^2^ Cage: 20 birds/m^2^	Hubbard, sexed groups	Economic analysis revealed that rearing meat chickens on the floor was more profitable than a cage rearing system. Data obtained in this experiment suggested that the welfare status of meat chickens was compromised under cage conditions as indicated by impaired performance, increased mortalities, higher prevalence of leg problems, stereotyped behaviour, and higher stress. In conclusion, cages were not recommended as a management system for rearing meat chickens from both economic and welfare perspectives.
14	Fortomaris et al. (2007) [25]	Behavioural and physiological parameters, ethogram	Performance and behaviour of broiler chickens as affected by the housing system	Germany; *n* = 870	(1) Straw deep litter (2) Cage	Litter floor pens: 15 birds/m^2^ Cage: 27 birds/m^2^	Cobb 500, sexed groups	There was more preening and wing flapping behaviour in litter systems.
15	Massey (2002) [26]	Physiological/body	Comparison of broiler breeder production and fertility in a colony cage system with two different floors versus a slat-floor system	USA; *n* = 870	(1) Slat + litter (2) Colony cage (3) Colony cage + cushion	Slat + litter: 5 birds/m^2^ Cage: 13 birds/m^2^	Cobb 500	Lower fertility was observed in the cage-maintained hens.

**Table 2:** Studies (8) suggesting *no* significant welfare problem using slatted floors or cages. Nb: If the material of the slatted floor was not stated, it was not reported. Stocking densities were not converted into consistent measures due to variable chicken body weights. WQA refers to Welfare Quality Assessment. Studies are sorted by publication date.

No.	Author(s); Date	Research Design	Title/Key Research Question	Sample				Key Result(s) and Recommendation
				Country; Sample Size	Floor/Housing Types	Stocking Density	Chicken Breed/Type	
1	Soliman and Hassan (2020) [27]	Environmental and bodily samples	Influence of housing floor on air quality, growth traits, and immunity in broiler chicken farms	Egypt; *n* = 200	(1) Wood shaving (2) Rise husks (3) Wheat straw (4) Plastic slats (5) Horizontal cages	Not stated	Hubbard	Slatted floors and battery cages were able to maintain indoor air quality, reduce microbial contamination, and enhance growth traits and immunity of meat chickens compared to traditional deep litter systems.
2	El-Deen et al. (2020) [28]	Physiological parameters	Effect of two housing systems on productive performance and some physiological traits of broiler chickens reared in enclosed houses	Egypt; *n* = 3120	(1) Cage (2) Litter—wheat straw, 5 cm	Cage—26 birds/m^2^, not exceed 58 kg/m² Floor—17 birds/m^2^	Ross 308	There was not much difference in blood parameters between two systems. Higher H/L ratio (i.e., stress) on floor system.
3	Çavuşoǧlu and Petek (2019) [29]	Welfare and behavioural parameters incl. RSPCA	Effects of different floor materials on the welfare and behaviour of slow- and fast-growing broilers	Turkey; *n* = 200	(1) Slatted floor (2) Deep litter—Rice hull	10 birds/m^2^	Slow-growing (Hubbard JA57) and fast-growing (Ross 308), males	Floor type did not affect behaviour parameters. Mean score of footpad dermatitis for the birds on the slatted floor was lower than for birds raised on deep litter at all ages. Meat chickens kept on the slatted floor were characterized by significantly lower hock-joint dermatitis scores throughout the experiment. No significant difference in gait scores. Slat flooring could be beneficial to improve meat chicken welfare, but further behavioural investigations are needed such as dust bathing and walking behaviour, i.e., covering more parameters.
4	Sargeant et al. (2019) [30]	Review/meta-analysis	The efficacy of litter management strategies to prevent morbidity and mortality in broiler chickens: A systematic review and network meta-analysis	International	n/a	n/a	n/a	There were no differences in mortality among the litter types, floor types, or additives. For footpad lesions, peat moss appeared beneficial compared to straw, based on a small number of comparisons. There was no association between fresh versus used litter on the risk of mortality, although there was considerable heterogeneity among studies. There was poor reporting of key design features in many studies, and analyses rarely accounted for non-independence of observations within flocks.
5	Suzer et al. (2019) [31]	Biomechanical bone characteristics	Effects of genotype and housing system on some bone biomechanical characteristics in broiler chickens	Turkey; *n* = 300	(1) Free-range –outdoor (2) Deep litter—7 kg/m^2^, unchanged (3) Plastic slat	Free-range: 10 birds/5 m^2^ Indoor: 10 birds/m^2^	Hubbard JA-57 SG and Ross 308 FG	Housing had no effect on bone characteristics.
6	Baracho et al. (2018) [32]	Review	Factors that influence the production, environment, and welfare of broiler chickens: A systematic review	n/a	n/a	n/a	n/a	Study did not find housing type to influence welfare.
7	Chuppava et al. (2018) [33]	Weight, footpad examination	Effect of different flooring designs on the performance and footpad health in broilers and turkeys	Germany; *n* = 720	(1) Litter (2) Litter + heat (3) Litter + partial slats (4) Slatted + sand bath, 900 cm^2^ Stored faeces. Slatted floor was plastic covered steel by Big Dutchman	35 kg/m^2^	Ross 308	The results in this study did not justify the use of slatted flooring systems. More research should be conducted to study the effects of slatted flooring on poultry welfare: behaviour, use of space, use of the sand bath, and other welfare indicators. Footpad dermatitis showed no difference between floor types. Slatted flooring might offer almost no possibilities for the birds to peck and manipulate particles when no litter particles are available on the ground, therefore, feed pecking occurs rather than pecking at the slatted floor resulting in higher feed intake. Insufficiently conclusive to be able to show whether litter floor pens with floor heating were superior to an entire floor pen without floor heating. “Other measures concerning behaviour, use of space, use of the sandbox, and other welfare indicators might have been very useful, but were not part of the study”.
8	Sunarti et al. (2010) [34]	Performance, physiological state, immune response	The effect of density and floor types on performance, physiological state, and immune response of broilers	Indonesia	(1) Litter (2) Bamboo slats	7/10/13/16 birds/m^2^	368 male broilers New Lohman strain	Link was found between stocking density and stress. It could be concluded that bamboo slats are best used for broilers up to a density of 13 meat chickens/m^2^.

**Table 3:** Welfare indicators assessed in the papers in Table 1 and Table 2. Nb: Performance/productivity indicators are excluded. Stars after names signify ‘et al.’. Some studies appear in more than one row or column.

Welfare/Behaviour/Physiological Parameter		Litter Floor Best	Slatted Floor Best	No Significant Difference
Injuries/lesions	Footpad dermatitis	Abd El-Wahab * [13] Almeida * [20] Almeida * [17]	Çavuşoǧlu * [18] Li * [19] Simsek * [22] Çavuşoǧlu * [29]	Chuppava * [33] Chen * [12]
	Hock burn	Almeida * [17]	Çavuşoǧlu * [18] Almeida * [20] Çavuşoǧlu * [29]	Li * [19]
	Breast blister/damage	Almeida * [17] Li * [19]	Çavuşoǧlu * [18]	Almeida * [20]
	Bruising (shank, drumstick)		Simsek * [22]	
	Other (fractures, bruises)	Simsek * [22]		Çavuşoǧlu * [18]
	Mortality	Fouad * [24]		Li * [19] Simsek * [22] Massey [26]
Blood/body measure	H/L ratio (high = stress)	Fouad * [24]	El-Deen * [28] Abo Ghanima * [14]	Sunarti * [34]
	Cortisol		Soliman * [27]	
	Oxidative stress biomarkers	Abo Ghanima * [14] Ozhan * [21] Simsek * [22]	Soliman * [27]	
	Antioxidant capacity		Soliman * [27]	
	LDH (biomarker for cell damage)		Abo Ghanima * [14]	
	Immunoglobulin IgG/IgM		Soliman * [27]	
	Bacteria counts		Soliman * [27]	
	pH of breast muscle	Ozhan * [21]		
	Glucose, uric acid, cholesterol	Ozhan * [21]		
	Other (e.g., lymphoid organs, bones, serum creatine kinase and alkaline phosphatase activity, CAT, fertility, ND antibody titter, oocyst)	Massey [26]	El-Deen * [28] Abo Ghanima * [14] Sunarti * [34] (oocyst, spleen)	El-Deen * [28] Süzer * [31] Abo Ghanima * [14] Ozhan * [21] Sunarti * [34] (bursa)
Behaviour	Gait	Almeida * [20] Li * [19] Almeida * [17] Fouad * [24]		Çavuşoǧlu * [18] Li * [19] Çavuşoǧlu * [29]
	Duration active	Fouad * [24]		
	Tonic immobility			Çavuşoǧlu * [29]
	Avoidance test/novel object test	Li * [19] Fouad * [24]		Çavuşoǧlu * [29] Li * [19] Chen * [12]
	Wing flap	Fortomaris * [25]		
	Panting			Chen * [12] Li * [19]
	Stereotypic behaviour (drinking)	Fouad * [24]		
	Comfort		Fouad * [24]	
	Aggression		Fortomaris * [25]	
	Preening	Fortomaris * [25]		
Dirtiness of plumage			Çavuşoǧlu * [18] Almeida * [20] Li * [19] Almeida * [17] Çavuşoǧlu * [29]	Chen * [12]
Feather condition			Çavuşoǧlu * [18]	
Air quality (CO_2_, ammonia)		Li * [19]	Soliman * [27] Almeida * [20] Almeida * [17]	Li * [19]
Expert view/review		El-deek * [16] Bracke * [15] Shields * [23]		Baracho * [32] Sargeant * [30]
Total		29	31	27

**Table 4:** Studies (10) suggesting means of enhancing welfare in litter floor systems. Nb: Stocking densities were not converted into consistent measures due to variable chicken body weights. WQA refers to Welfare Quality Assessment. Studies are sorted by publication date.

No.	Author(s); Date	Research Design	Title/Key Research Question	Sample				Key Result(s) and Recommendation
				Country; Sample Size	Floor/Housing Types	Stocking Density	Chicken Breed/Type	
1	Adler et al. (2020) [35]	WQA	Effects of a partially perforated flooring system on animal-based welfare indicators in broiler housing	Germany; *n* = 500	(1) Partially perforated platforms (polypropylene-based perforated elements) (2) Littered	39 kg/m^2^	Fast-growing 308 Ross	Results showed that the partially perforatedflooring system had a positive influence on footpad dermatitis from day 14, and hock burn on day 28. There was no effect on production performance.
2	Fidan et al. (2020) [36]	Welfare criteria examination	The effects of perch cooling on behavior, welfare criteria, performance, and litter quality of broilers reared at high temperatures with different litter thicknesses	Turkey; *n* = 459	(1) Perches (none, non-cooled, cooled) (2) Litter thicknesses (1, 7, 14 cm)	10 birds/m^2^	Male, day-old	Cooled perches and 14 cm of litter thickness tended to decrease the incidence of footpad dermatitis and hock burn. The body weight gain of the broilers in the cooled perch group was higher than those in no perch and non-cooled perch groups. These results suggest that cool perches have a beneficial effect on the performance and welfare of broilers.
3	Freeman et al. (2020) [37]	Physiological and injury measures	Remedying contact dermatitis in broiler chickens with novel flooring treatments	USA; *n* = 546, 42 pens, 13 per pen	(1) Plastic slats + litter (2) Disinfectant mats + slats + litter (3) Used litter (4) Clean litter	10.4 chicks/m^2^ at placement; stocking density of 35.8 kg/m^2^	Hubbard X Ross	Unexpectedly, the positive control, consisting of replacing litter every four days, resulted in the best welfare condition (footpad dermatitis, hock burns, and gait); the other flooring types did not remedy or prevent contact dermatitis. This comparable approach may be commercially feasible.
4	Pedersen et al. (2020) [38]	Post-mortem analysis	Effects of environmental enrichment on health and bone characteristics of fast-growing broiler chickens	Denmark; 497 birds in each of 58 pens	Deep litter system with trials of different enrichments such as: (1) Two distances between food and water (2) Platforms (3) Lower density 34 kg/m^2^ (4) Opaque vertical panels	40 kg/m^2^	Fast-growing Ross, both sexes, day-old	Provision of vertical panels and increased distance between resources can result in larger muscle and bone dimension, possibly having a positive effect on leg health. Furthermore, the provision of environmental enrichment does not appear to be a risk factor for wooden breast or bacterial infection.
5	Meyer (2019) [39]	Behavioural and physiological measures	A novel environmental enrichment laser device stimulated broiler chicken active behavior and improved performance without sacrificing welfare outcomes	USA; *n* = 1260	Litter with laser Litter without laser	Not stated	Ross 308, day-old	Lasers improved welfare and weight gain. Good for commercial environments.
6	Riber et al. (2018) [9]	Review	Review of environmental enrichment for broiler chickens	n/a	Non-cage options	n/a	n/a	Many of the ideas for environmental enrichment for broilers need to be further developed and studied, preferably in commercial trials, with respect to the use, the effect on behaviour, and on other welfare aspects such as leg health, and the interaction with genotype, production system, stocking density, light, and flock size.
7	Kaukonen (2017) [40]	WQA	Housing conditions and broiler and broiler breeder welfare	Finland	Litter variations—platforms, peat, and more	42 kg/m²	Fast-growing broiler breed, Ross 508	Regarding footpad health, peat seems to be the optimal litter material for Finnish conditions. Farmer ability to manage litter conditions is important, regardless of the chosen litter material. Hock burn monitoring could represent a more sensitive indicator of litter condition and possibly also signal leg health status. Platforms should be preferred over perches as the latter are unused. Platform availability could enhance broiler wellbeing.
8	Mesa et al. (2017) [41]	Physiological measures	Assessing the effects of different housing conditions on both feed conversion ratio and mortality of male broiler flocks	Brazil; >100 million birds, 977 farms, one major producer, 3516 flocks	Concrete +litter Dirt	12 birds/m^2^	Male Ross 308, one-day	Positive ventilation, metal and clay roof, dirt floor, and owner management were shown to reduce mortality.
9	Kaukonen et al. (2016) [42]	WQA	Effect of litter quality on footpad dermatitis, hock burns, and breast blisters in broiler breeders during the production period	Finland; 10 farms, 18 houses	Slatted litter	6 birds/m^2^	Ross 508, 10% male	The condition of footpads deteriorated towards slaughter age, with the occurrence of severe lesions reaching a maximum of 64% on average at slaughter. Hock lesions and breast blisters were rare. The litter layer became drier over time. Although poorer litter condition and wetness influenced footpad health negatively, the effect on severe lesions was not significant. A negative effect on footpad condition of larger slat areas was observed. In conclusion, maintaining good litter quality alone is not enough to ensure healthy footpads.
10	Simsek et al. (2009) [43]	Physiological measures	Effects of enriched housing design on broiler performance, welfare, and serum cholesterol	Turkey; 480	Litter with perches/sand Litter without (wood shavings)	15 birds/m^2^	Ross 308, sexed groups	Housing enriched with perches and sand bedding in addition to wood shavings improved broiler welfare and meat quality.

## Data Availability

Not applicable.
